# Sudden onset chest pain after a CT-scan of the aorta

**DOI:** 10.1007/s12471-024-01913-4

**Published:** 2024-12-09

**Authors:** Fabienne E. Vervaat, Thomas van Brakel, Sjoerd Bouwmeester

**Affiliations:** 1 Department of Cardiology, Catharina Hospital, Michelangelolaan 2, 5623EJ Eindhoven, The Netherlands; 2 Department of Cardiothoracic surgery, Catharina Hospital, Michelangelolaan 2, 5623EJ Eindhoven, The Netherlands

A 73-year-old woman presented at our emergency department with sudden chest pain. Her medical records showed a previous diagnosis of bicuspid aortic valve with secondary aortic root (45 mm) and ascending aorta (47 mm) dilatation in 2014, as well as hypertension, which was controlled with metoprolol 50 mg OD and lisinopril 20 mg OD. Since then, she had been undergoing annual CT scans or transthoracic echocardiograms for routine follow-up.

In 2021, her ascending aorta measured 49 mm on CT, while the aortic root remained at 47 mm, and the bicuspid aortic valve showed normal function on echocardiogram. By 2023, the maximal ascending aorta diameter had increased to 58 mm (Fig. 1). Six hours after the CT scan, the patient experienced sudden chest pain, prompting immediate medical attention. Upon presentation, she was conscious and responsive, with a blood pressure of 122/44 mm Hg, heart rate of 58 bpm, oxygen saturation of 100% without supplemental oxygen, capillary refill time of 3 s, and normal physical examination.

**Fig. 1:**
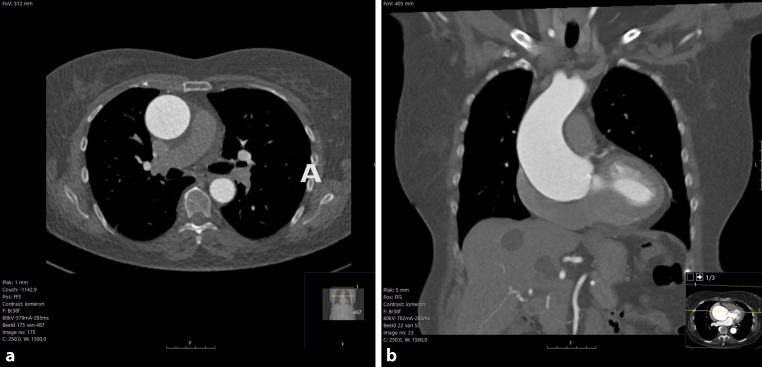
**a**, **b** CT-scan of the aorta prior to symptom onset with dilated aortic root and ascending aorta

What is the diagnosis?

## Answer

You will find the answer elsewhere in this issue.

